# *“We really are seeing racism in the hospitals”*: Racial identity, racism, and doula care for diverse populations in Georgia

**DOI:** 10.1371/journal.pone.0286663

**Published:** 2023-06-07

**Authors:** Ayeesha Sayyad, Alyssa Lindsey, Subasri Narasimhan, Daria Turner, Priya Shah, Ky Lindberg, Elizabeth A. Mosley

**Affiliations:** 1 Health Promotion and Behavior Concentration, School of Public Health, Georgia State University, Atlanta, GA, United States of America; 2 Department of Behavioral, Social and Health Education Sciences, Center for Reproductive Health Research in the Southeast, Rollins School of Public Health, Emory University, Atlanta, GA, United States of America; 3 Hubert Department of Global Health, Rollins School of Public Health, Emory University, Atlanta, GA, United States of America; 4 Healthy Mothers Healthy Babies Coalition of Georgia, Atlanta, GA, United States of America; 5 Department of General Internal Medicine, School of Medicine, University of Pittsburgh, Pittsburgh, PA, United States of America; Federal University of Sao Carlos: Universidade Federal de Sao Carlos, BRAZIL

## Abstract

**Introduction:**

Poor birth outcomes are more prevalent for Black communities, but strong evidence shows that doula care can improve those outcomes. More evidence is needed to understand racial differences, discrimination, and equity in doula care.

**Methods:**

The current study’s objective was to describe the experiences of Black doulas as well as the challenges and facilitators of providing doula care to communities of color in Georgia. From Fall 2020-Fall 2021, 20 surveys and in-depth interviews were conducted with doulas as part of a community-based participatory study co-led by Healthy Mothers, Healthy Babies Coalition of Georgia and academic researchers.

**Results:**

Doula participants were diverse in age (5% under 25, 40% 25–35, 35% 36–45, and 20% 46+) and race/ethnicity (45% white, 50% Black, 5% Latinx). Most (70%) Black doulas reported that more than 75% of their clientele is Black, while most (78%) white doulas reported that less than 25% of their clientele is Black. Doulas noted the alarming Black maternal mortality rate and how mistreatment causes Black clients to lose trust in medical staff, leaving them in need of advocates. Black doulas were passionate about serving and advocating with Black clients. Participants also described how language and cultural barriers, particularly for Asian and Latinx people, reduce clients’ ability to self-advocate, increasing the need for doulas. Doulas also discussed the ways that race influences their connections with clients and their dissatisfaction with the lack of cultural humility or sensitivity training in standard doula training.

**Conclusion:**

Our findings indicate that Black doulas provide essential and supportive services to Black birthing people, and those services are more urgently needed than ever following the overturn of *Roe v*. *Wade*. Doula training must be improved to address the cultural needs of diverse clients. Increasing access to doula care for Asian and Latinx communities could also address language and cultural barriers that can negatively impact their maternal and child health outcomes.

## Introduction

Maternal mortality—the death of a person from a pregnancy related cause—is a major health issue globally that disproportionately affects women of color. The United States (US) has a very high mortality rate when compared with other high-income developed countries [1] and maternal mortality is increasing in the US while declining globally [2]. Within the US, the maternal mortality rate is worst for non-Hispanic Black women, who have a maternal mortality rate that is more than three times the rate for white women [3]. This disparity is also present for infant deaths, with non-Hispanic Black infants two times more likely to die than non-Hispanic white infants [4]. To-date, perinatal quality improvement initiatives have largely erased the experiences of Black women, and they have failed to close the racial gaps or address the need for cultural rigor [5]. The state of Georgia has been identified by the Centers for Disease Control and Prevention as having one of the highest infant and maternal mortality rates in the country [6]. The racial disparities in birth outcomes seen nationally are also present within the state of Georgia, with Black mothers in the state being three times more likely to die from a pregnancy-related cause than white Georgia mothers [7]. Moreover, maternal deaths are only “the tip of the iceberg,” [8]—for each life lost, there are many others who experienced morbidity or a negative and potentially traumatizing birth.

Doula care has been shown to improve birth experiences and outcomes, including higher satisfaction, less likelihood of preterm birth, less likelihood of low birthweight, and increased likelihood of vaginal delivery [9]. Doulas are non-medical birth support personnel, who provide physical and psychosocial comfort to birthing people [9]. The continuous birth support they provide has been shown to reduce unnecessary medicalization of births and ultimately improve birth outcomes and maternal and infant health [10]. There is evidence that doula care has benefits specific to marginalized women, who are most at risk for poor birth outcomes, including Medicaid patients and Black birthing people [11]. For example, a 2016 study by Kozhimannil et al. found that doula care significantly reduced cesarean sections and preterm births in Medicaid beneficiaries [12]. Reductions in cesarian sections can be linked to reductions in severe maternal morbidity, lowering the risk of maternal mortality [13,14]. Other studies in North Carolina and Minneapolis have shown that Black mothers with doulas had fewer birth complications, less risk of low birthweight babies, and greater likelihood of breastfeeding than Black mothers without doulas [15–18]. One qualitative study with 13 racially/ethnically diverse mothers living on low incomes in Minneapolis found that culturally concordant doulas (i.e., Black doulas from lower socioeconomic communities) act as protectors from negative social determinants of health—the predisposing factors that influence health outcomes, like racism and income level [15]. The study participants detailed how the support and advocacy that doulas provide increased their agency, personal security, knowledge, respect, connectedness, comfort, and self-efficacy during birth, thus facilitating a “Good Birth” experience [15].

Yet while Black and lower income women stand to benefit greatly from doula care, there are major barriers to access [16–18]. For lower income birthing people on public Medicaid insurance, very few states offer doula reimbursement [15]. Moreover, a 2014 study of 2,400 women who had recently given birth in the U.S found that Black women were more likely than white women to want a doula but not have one [18]. Another study conducted at three midwestern health clinics found that Black women were less likely than white women to have heard of doulas; this difference was greatest between wealthy white women and low-income Black women [17]. Additionally, doulas in New York City reported that they struggle to find clients who can afford their services, leading them to serve few lower income women [16].

These barriers to doula care for Black and lower income communities is not for lack of desire and passion among doulas from those communities. Numerous qualitative studies have demonstrated that Black doulas are passionate about serving Black clients [19–21]. Hardeman and Kozhimannil interviewed 12 doulas of color in Minneapolis and found doulas were passionate about providing culturally competent care to members of their communities [19]. In other studies, Black doulas have described their commitment to social justice and how they used a culturally centered approach to meet the needs of their clients [20]. A 2019 study with Black doulas identified that low pay is a major barrier [21]. Black doulas expressed a desire to mostly serve Black clients but faced financial limitations, because Black clients were less likely to be able to afford out-of-pocket doula care, leaving many Black doulas working multiple jobs to supplement their doula income [21]. This is a major public health challenge, because Black doulas are essential to providing culturally competent doula care to Black mothers given their shared lived experiences, cultural values, and health concerns [19,21–24].

The aforementioned studies on race, income, and doula care were primarily centered in the midwestern United States, with only one having participants from the Southeast [11]. Further research is needed to explore the experiences of Black doulas in the Southeast, where some of the worst birth outcomes in the country are found [4,6]. Additionally, prior studies that include doulas as participants have been mostly qualitative and not quantitative, so further quantitative research on doula care from the perspective of doulas is especially needed. Finally, few studies on doula care have utilized a community-based participatory research approach. Community based participatory research on doula care is critical because it centers the needs of the community in all phases of a research project [25]. Community-based participatory studies on doula care can center marginalized groups, who are often left out of research but are most vulnerable to poor birth outcomes [3]. A community-based participatory approach requires that researchers share power with the community being researched and affected by the negative health outcomes, which can increase buy-in from community members and increase community capacity to address public health issues [25]. For these reasons, community-based participatory doula research is needed to empower communities of color, lower income communities, and other marginalized groups at higher risk of poor birth outcomes to advocate for their access to doula care.

### Research questions and objectives

The aim of this study is to explore doula’s views and perceptions about caring for communities of color. The research questions addressed in this study were:

1. What are the experiences of doulas serving clients of color, including doulas of color and white doulas?
2. What are the challenges and facilitators of Black doulas when providing doula care?
3. What communities do Black, white, and other doulas serve and what are their motivations?

## Materials and methods

The Georgia Doula Study is co-led by community-based organization Healthy Mothers Healthy Babies Coalition of Georgia (HMHBGA) and an academic researcher, who is also a full spectrum doula. The Georgia Doula Access Working Group, first convened in 2019 to improve access to doula care for all Georgians, served as the Community Advisory Board for this study. The Georgia Doula Access Working Group has representation from health professionals, doulas, researchers, policy makers, and community leaders. The purpose of the advisory board was to ensure stakeholder engagement in the development of study instruments, assist with recruitment, review preliminary data to assist with interpretation, and facilitate dissemination of findings back to doulas, hospital systems, insurers, and policy advocates. The study team consisted of two academic researchers (EAM, SN), a lead graduate student researcher (DT), three Graduate Research Assistants (AS, AL, PS), and a community partner from HMHBGA (KL). The graduate student researchers received weekly training in qualitative data collection, qualitative data analysis, community-based participatory research, and reflexivity from EAM.

The community-academic research team designed and conducted a cross-sectional mixed methods observational study interviewing and surveying doulas in metro-Atlanta Georgia. Participants were recruited through emails to the Georgia Doula Access Working Group. All doula members of the working group were encouraged to participate and to share the opportunity to participate in the study with their networks. The study procedures were reviewed by the Emory University Institutional Review Board and deemed exempt from IRB oversight because the human subjects could not be easily identified, and any disclosures would not place subjects at risk of damages [see rule 45 CFR 46.104(d)(2i(2ii)]. Verbal consent was obtained from all participants. Participants were given $20 for their participation in the study. Inclusion criteria were being over 18 years of age, self-identifying as a doula, having worked as a doula in Georgia for at least 6 months, and proficiency in English. The surveys covered doula demographics, client demographics, doula practice, changes to services during COVID, doula reimbursement, and beliefs about doula services. The interviews included questions regarding doula training, practice, clientele, doula reimbursement, client stories and challenges to providing care. These semi-structured in-depth interviews were conducted to collect more detailed information on survey domains as well as to elicit information regarding client stories and challenges to providing care. In the fall of 2021, additional measures on racism and discrimination in doula care were added to the interview guides, and previous participants were re-contacted. Participants were given an additional $20 for completing the additional survey and interview.

The surveys were analyzed using descriptive and bivariate statistics in Microsoft Excel and Stata v 14 [26]. Interviews were transcribed verbatim, de-identified, and thematically analyzed using coding, memo-ing, and diagramming in the online qualitative software Dedoose [27]. For each interview, the interviewer identified ten inductive topics of greatest importance for that participant. These inductive topics were combined with deductive codes already identified by the research team (e.g., domains from the survey and interview guide) to form a codebook. Each transcript was coded by two researchers in Dedoose, who met to discuss any discrepancies in coding until they reached consensus. Analytic memos were created for each code, including those highlighted in this manuscript such as discrimination, underserved populations, client stories, relationship with clients, and neglect of patient autonomy. The team met bi-weekly to discuss emerging themes across the codes and differences in those themes across groups (i.e., across racial groups).

## Results

The research team originally surveyed and interviewed 17 doulas between October 2020 and February 2021. In the fall of 2021, fourteen previous participants were re-interviewed; and three additional participants were interviewed and surveyed for a total of 20 doulas. Participant demographics are presented in Table 1. The 20 doula participants were 40% white, 45% Black, and 5% Latinx. Participants were diverse in age with 40% between 25 and 35, 35% between 36 and 45, and 20% 46 or older. Most (70%) of the doulas had at least a bachelor’s degree, while 30% had less than a college degree. The majority (85%) of the doulas were not immigrants and, similarly, 80% identified as heterosexual.

**Table 1:** Demographics of the doula sample (n = 20).

Variable	Frequency	Percent
**Race/Ethnicity** ** **Black or African American ** **White ** **Hispanic or Latinx ** **Black or African America, Other ** **White, Other	9 8 1 1 1	45% 40% 5% 5% 5%
**Gender Identity** ** **Cis-gender Female ** **Nonbinary or Genderqueer	18 2	90% 10%
**Age** ** **Under 25 ** **25–35 ** **36–45 ** **46–55 ** **Over 55	1 8 7 3 1	5% 40% 35% 15% 5%
**Economic Status** ** **Prefer not to say ** **Currently experiencing economic difficulty ** **Experienced economic difficulty in the past ** **Experienced economic difficulty historically ** **Experienced economic difficulty temporarily ** **Never experienced economic difficulty	1 1 1 2 5 10	5% 5% 5% 10% 5% 50%
**Education** ** ** Some college/technical degree ** **Non-clinical professional degree ** **Graduated college ** **Clinical professional degree ** **Graduate degree	4 2 9 2 3	20% 10% 45% 10% 15%
**Employment** ** **Yes, full-time ** **Yes, part-time ** **No, not looking for employment ** **No, looking for employment	12 3 3 2	60% 15% 15% 10%
**Sexuality** ** **Straight/heterosexual ** **Bisexual ** **Queer ** **Lesbian	16 1 2 1	80% 5% 10% 5%
**Primary Language** ** **English ** **Portuguese ** **Jamaican Patois	18 1 1	90% 5% 5%
**Immigration Status** ** **Not an immigrant ** **First generation immigrant	17 3	85% 15%
**Ever Been Pregnant**	15	75%
**Had a Doula Personally**	5	33%

The percentage of doula clientele that were Black are presented in Table 2 by race of the doula. The majority (70%) of Black doulas reported that over 75% of their clients are Black. This is contrasted with the majority (78%) of white doulas reporting that less than 25% of their clients are Black.

**Table 2:** Percentage of clients who are black among the doulas surveyed and interviewed (n = 20).

Race of the Doula	<25%	25–75%	>75%
Black Doulas (10)	1 (10%)	2 (20%)	7 (70%)
White Doulas (9)	7 (78%)	2 (22%)	0

### Doulas shared their experiences with medical racism through client stories

Doulas commonly described experiences of medical racism when describing the experiences of their Black clients. One doula gave an account of her Black client’s autonomy being disrespected while the doctor neglected to even introduce themself before touching the patient.

*“I remember I literally witnessed a white doctor walk in and*.. *not say a word*, *literally looked at the monitors and was*…*going to grab my client*. *Literally…*. *We did not find out who it was*. *I literally ran around and like*, *‘are you the doctor*?*’ And then the doctor was like*,*’ yeah’…So then they go to grab the suction cup to put it on the baby head without even asking*. *So this is where the doctor probably got mad at*, *because I said*, *‘hold on*, *you need to get consent’*. *Did you get consent from her*? *What is going on*? *She’s asking you what’s going on*… .*So the doctor*, *the baby comes out*, *the doctor grabs a needle to numb her*…*So I’m like*, *I know what’s going on*. *So*, *I stopped and I’m like*, *‘did you*, *did you want sewing*?*’…mind you-we’d never heard that she’d even tore*, *nothing*. *Didn’t say anything to her*. *I mean the whole entire time that doctor has not spoken and the baby is out*, *the baby is already on her chest”-*Nicole^1^, Full Spectrum Doula

Another doula told the story of a Black client who had struggled with substance abuse being mistreated by a social worker and their physician. The doula noted that clients of color often have surprise visits from social workers, something that is always bothersome, but had particularly poor consequences for this client.

*“So*, *a social worker came by when they were in the hospital after they had their baby… you know… the random social worker check that happens so often for people of color… but basically the social worker had outed my client for being in recovery from substance use disorder and was treating them like they were*, *for lack of a better word*, *like a junkie*… .*Like*, *this person outed them to their provider and then their provider started treating them funny*.*”-*Bailey^1^, Full Spectrum Doula

Another doula shared that she has seen providers behave differently based on a client’s race.

*“And then like I’ve just seen*, *I’ve seen like one provider treat a white patient and then I’ve seen like one provider to a Black patient and it’s different*. *You know they’re not talking quite as much to them maybe they’re not in the room*, *quite as much they’re not touching them as much*, *they’re not doing as much eye contact*, *like their explanations are very different*, *like one was like very dumbed down and like one was like talking to like their equal so I’m not even though they were talking*, *they were explaining it in a deeper way where*, *like the other*, *like the other time it was just like not explained well at all*. *And I’m like*, *this is very different*.*”-* Annie^1^, Birth and Postpartum Doula

### Medical racism causes distrust among black birthing people

Doulas described how Black clients experience racialized mistreatment in the medical system. Not only have many Black clients had these experiences in the past, those who have not are aware that they are susceptible to mistreatment. This causes a great deal of mistrust.

“*Well*, *let’s just start with the Black community*. *I mean we really are seeing racism in the hospitals and there’s a huge divide in the way a white Caucasian person going in*, *that’s pregnant and complaining of chest pains or something versus if you had a Black woman who’s complaining that and really just like being ignored or not taken seriously like that is not okay*.”-Alicia, Birth and Postpartum Doula

Doulas also gave details on how the high maternal mortality rate for Black mothers causes Black clients to see doula care as a necessity. Doulas noted that Black clients see themselves as vulnerable to mistreatment and poor birth outcomes and believe doulas can provide protection.

*“Early in the pandemic*, *I would say that I lost quite a few of the clientele that I had*, *um*, *until*, *and this is very unfortunate to say*, *until that Black maternal [mortality] rate started going up*. *And it seemed like every week*, *a Black mom was dying in childbirth*. *When*, *when a lot of my clients were seeing that or just anybody on my social media*, *that’s when they kind of started noticing*, *okay*, *I need more support in the hospital than just my partner that knows nothing about birth*, *or just my mom*.*”*–Brianna (pseudonyms are used throughout to protect participants’ confidentiality), Full Spectrum Doula

Black clients’ mistrust of the medical system has led many to feel it is safer to give birth outside of the hospital. Additionally, Black clients desire a non-judgmental kind of support they feel uncertain they will receive from medical birth support personnel in a hospital.

*“That’s a change in maternal mortality*, *like every time I get a family*, *especially families of color choosing to birth at home and hire birth support at home*. *That is the biggest impact because we know the highest death rate for people of color is birthing in the hospital*. *So*, *every time that happens*. *It’s a huge impact*. *Like*, *man*, *I’m just grateful that every time I get to*……. *work with our families of color*. *You get to see the impact by just being able to support them and then having somebody that’s not trying to tell them what to do*, *but just really provide them with those tools that we oftentimes don’t get unless we get support*.*”*- Nicole^1^, Full Spectrum Doula

### Race impacts the connection doulas have with their clients

As noted above, survey responses indicated that doulas are mostly serving clients of their same race. Interview responses gave insight into possible causes of this trend. A white doula shared that she noticed some Black clients being uncomfortable with her touch.

*“I think that there’s definitely been times where I may have had to work a little harder to make some of my Black clients feel comfortable about you know me being the one that’s touching all over them*, *providing that physical support*.*”*-Jessica^1^, Birth and Postpartum Doula

Another white doula noted that her race may make her a poor fit for certain potential clients.

*“I’m not the right doula for everybody*, *I’m just not right because of that type of connection*. *You know*, *and I think that’s what’s also so neat about this job is like*, *there is a doula out there for everybody*, *you know*, *and I do I have started to say that to in certain consults when I don’t feel like I can serve them the best way*.*”*- Sarah^1^, Birth Doula

Conversely, one Black doula described her own desire to have care providers of the same race and shared that she believes her clients have the same desire.

*“I believe most people want to receive care from people who resemble them who look like them*, *and who know their specific struggles and needs within their own community…I know for me I would want somebody who looked like me who may have experienced things that I’ve experienced*.*”–*Jasmine^1^, Full Spectrum Doula

### Black doulas are passionate about and focused on serving black clients

When asked what communities they would like to serve, Black doulas expressed their passion for serving and advocating with Black clients.

*“Um definitely [want to be a doula for] black birthing people*, *post*, *Black postpartum people*.*”*-Imani^1^, Full Spectrum Doula

In their survey responses, 86% of Black doulas reported that more than 75% of their clients were Black. In their interview responses, Black doulas expressed satisfaction with being able to do most of their work with Black clients.

*“My demographic was Black and African American*,… *So*, *I’m grateful for being able to serve my community*, *the community that I specifically when out to serve*.*”*-Nicole^1^, Full Spectrum Doula

Black doulas also described their passion for making sure that Black birthing people have access to doula care. This led some Black doulas to expand on their role as doulas and become advocates for policies that promote access to doula care.

*“Me being a Black doula…I am specific to making sure that I am advocating and I’m you know staying up to date on legislation and politics and how that all affects access to having a doula and the level of concern that your elected officials have for the fact that mothers*, *especially Black mothers are dying*, *or Black people are dying when they’re giving birth*. *So*, *my advocacy comes in the form of not really being hired as an advocacy doula*, *but the advocacy is a part of being a doula and then just the justice part of it*.*”*-Imani^1^, Full Spectrum Doula

### Doulas know their black clients are at risk, and have mechanisms to protect them

Several doulas shared that they feel the need to protect Black clients from mistreatment in the hospitals. One doula encouraged her clients to think about the environment they will be birthing in when picking providers and birthing facilities.

*“I encourage my Black clients to consider where they’re delivering and who their providers are*. *Are there any people of color in that practice*? *How about the nurses at the hospital*? *Are you going to go to an all-white hospital*? *All-white staff and you don’t see a lot of*, *you know*, *Black people*..?*”-* Annie^1^, Birth and Postpartum Doula

Another doula described her way of humanizing her clients in an attempt to dissuade hospital staff from being discriminatory.

*“So*, *I tried to definitely humanize them for the staff*, *their doctor*, *anybody coming in the room*, *to ensure that your biases are kind of checked at the door and that you’re going to treat this person like an individual*, *not a color*, *not an age*, *not a demographic*, *but just a human who you need to treat with empathy and do no harm to*. *And I guess that kind of curbs that*, *but I do see it*. *I see the discrimination*. *I see the lack of care*. *But*, *like I said*, *I get to it right away*. *I’m going to fix that problem right away*, *because if I don’t*, *that could lead to them having hemorrhaging or preeclampsia or not being looked after*, *not being cared for and often missing something*.*”-* Imani^1^, Full Spectrum Doula

There was also a doula who worked with her clients to prepare them to identify mistreatment as it is happening.

*“So when certain things go down in the hospital where discrimination has happened*, *and I know what my steps are*, *you know*, *for my families and stuff like that*. *And so we go over that*. *So that way we are prepared*, *you know*, *with our training to be able to recognize within certain language*, *you know*, *I have taught my families about how to hear how they speak to you*.*”-*Nicole^1^, Full Spectrum Doula

### Asian and Latinx Birthing people have specific cultural needs and language barriers doulas can address

Doulas also described how their clients with language barriers greatly appreciated their advocacy and needed someone to relay information to the medical team. The following quote is from a doula with a Japanese speaking client, who struggled with English. The doula did not speak Japanese but was able to work with the client and understand that her birth plan included keeping her placenta. The medical team did not understand the client and the doula was able to step in and advocate for her client.

“I think with when she seen that I was able to stand up for her advocate for her even though she knew that they wasn’t understanding anything that she was trying to say. But she knew that I did. She ended up writing me up an awesome review, um in Japanese on social media and posting it and it was in Japanese, so I had to translate it, and it was just absolutely beautiful. I can just tell that she was just so thankful for me being there.”

*-*Brianna^1^, Full Spectrum Doula

Doulas understood that birthing people from immigrant communities may not have access to their extended family networks, leaving them in need of additional birth support.

*“I would say that the Latin culture is very big on family and having that familial support and I would say that Latin women who have come here without all of their family; there would be that huge gap in kind of that support and that’s what I would like to provide*. *If they can’t have a sister or a mom there to support them*, *I would like to be in that role just for a short time and be able to support them emotionally*.*”*-Jessica^1^, Full Spectrum Doula

All of the doulas reported that less than 15% of their clientele was Asian or Latinx. When asked what communities they would like to serve that they have yet to reach, doulas frequently answered Asian and Latinx communities. Doulas also described how language and cultural barriers reduce Asian and Latinx clients’ ability to advocate for themselves, increasing the need for doulas.

*“Um*, *I definitely want to get more into trying*, *being able to train to work for the Latino community because I feel like they definitely need like advocates for them there*. *And I would love to be a doula for them*. *So*, *what I’m working on right now is*, *before I moved to Georgia*, *I was very fluent in Spanish*. *However*, *I didn’t use it*, *so I lost it*. *I still have some key words that I understand*, *but there are some where I’m still like*, *‘uh*, *let me look this up on the phone’*. *So*, *right now*, *I’m focusing on trying to find a program that is going to allow me to learn a little bit more Spanish so that I could go out to the Latino community and be able to be of service to them as well*.*”* -Andrea^1^, Birth and Postpartum Doula

### Doula training is not adequately addressing specific racial and cultural needs

When asked about the training they received about providing culturally competent care, doulas mostly said it was insufficient. One doula said her training did not address race or culture at all.

*“And they don’t talk about it at all*. *Through like my [Doula Training Organization] training which is like the largest international training organization*. *I don’t even think it’s touched on*.*”-*Annie^1^, Birth and Postpartum Doula

Another doula discussed how her training focused on diversity rather than the biases that specific groups are likely to face.

*“I didn’t learn about medical biases until actually becoming a doula…you’re going to get more along the lines of inclusiveness training*, *which is more like everybody should be treated fair*. *We don’t want to discriminate*, *which as we know glazes over [the real issues]*.*”-* Nicole^1^, Full Spectrum Doula

There was also a doula who noted that more progressive doula trainings are often less respected.

*“So*, *you know there’s sometimes that little discrimination where I feel like if you’re not DONA certified*, *it’s not good enough sometimes*. *I get that vibe*. *But I’m hoping that that changes as [other doula training organizations] get more notoriety and other certifying entities come about with the same progressive type of curriculum*.*”*-Imani^1^, Full Spectrum Doula

## Discussion

This was a community-engaged mixed methods study investigating doula care for communities of color in metro-Atlanta, GA. These results further our understanding of how culturally appropriate doula care, (particularly from doulas who share the racial identity of their client) can reduce maternal health disparities [22,28]. Doulas shared the medical and obstetric racism they have witnessed, while serving their clients of color in hospitals. They detailed how these experiences of racism have caused their clients to be distrusting of the medical system. Doulas also described how their race can impact the connection they can form with their clients of the same race and of other races. Black doulas, specifically, were passionate about serving Back clients and addressing disparities in their maternal health. Doulas of all races described a strong desire to protect their Black clients from any possible discrimination. Asian and Latinx communities were identified as having specific cultural and language needs that doulas can address. Doulas also described how the training they received was not always sufficient in preparing them to meet the cultural needs of their clients.

Black doulas’ explicit desire to serve and advocate for Black clients supports findings from a previous study in Minneapolis that suggested doulas can protect clients from the negative social determinants of health, like racism and poor economic stability [15]. Our Black doula participants wanted to shield their clients from mistreatment through education, respect, and support. Our findings also point to issues facing Black patients that have been previously discussed in the literature. Davis has used doula client stories and other data to describe how obstetric racism negatively impacts Black birthing people’s experiences and contributes to poor birth outcomes [22]. Additionally, race concordant care has been shown to improve trust and communication, especially for Black patients [23]. These findings align with what our participants described about how race can impact the connection they form with their clients. Lastly, cultural humility trainings have been developed for various kinds of clinicians and there is some evidence that they improve patient experiences, though more research is needed [24]. Our doulas described a desire to be better trained to meet the cultural needs of their clientele, cultural humility trainings used outside of maternal health could be adapted to be applied to doula care.

Our survey data suggests that Black clients in metro-Atlanta, Georgia are mostly being served by Black doulas. Previous research has pointed to financial issues that doulas encounter when serving mothers from marginalized groups [16]. Our findings suggest that this burden is mostly being carried by Black doulas. This means that Black doulas also stand to benefit the most from programs that provide financial assistance to birthing people, who cannot afford doula care. Studies suggest that Medicaid coverage for doula care would significantly improve health outcomes and ultimately reduce Medicaid spending [16].

Our findings regarding doula care for Latinx and Asian clients suggest that these communities could benefit from expanded access to doula care. Particularly, there is a need for bilingual doulas to service clients with varying levels of English proficiency. In our study, there was a doula that was able to assist her Japanese speaking client without speaking Japanese. This situation resulted in a positive outcome for the client, but it would have been ideal for the client to have had a Japanese-speaking doula. In 2012, researchers reviewed a program at a midwestern hospital, where doulas were bilingual and acted as translators, and found that it had positive effects on birth experiences and outcomes [29]. The program was well-received by both patients and medical providers, because it facilitated communication between the Spanish-speaking patients and mostly English-speaking staff [29]. Similar programs may be helpful if implemented in other areas with communities that experience language barriers.

The primary strength of this study is the community-based participatory approach, and the primary limitation is the small sample size. The study is co-led by Healthy Mothers Healthy Babies Coalition of Georgia and was designed with input and oversight from the Georgia Doula Access Working group. This gave doulas, health professionals, researchers, policy makers, insurance payers, and community leaders the power to design the research questions, execute the study, increase community buy-in, and facilitate dissemination of findings. The sample size of 20 did not allow for statistically meaningful inferential statistics to be performed. Other limitations include the lack of inclusion of rural and immigrant doulas and the inclusion of only doula perspectives. The doula perspective was amplified in this study because of the lack of studies from doula perspectives currently present in the literature, but future studies could also interview diverse doula clients.

This current study has several implications for future research, practice, and policy—particularly as access to abortion and other comprehensive reproductive health services are severely eroded with disproportionate consequences for Black and other communities of color [30,31]. First, more community-engaged research with diverse doulas in high-risk settings like the Southeast is needed to improve understanding about the challenges and facilitators of doula care for marginalized communities [32]. Future studies might also include quantitative measures of racism against doulas in a larger, more representative sample, and could incorporate client experiences of obstetric racism as measured by the PREM-OB scale [5]. Second, increased training opportunities are needed for doulas of color—especially Black, Latinx, and Asian doulas—in order to provide culturally-appropriate, full spectrum doula care for their communities. These trainings need to be free, must include support for building doulas businesses, and provide opportunities for mentorship and networking. This is one way to work toward cultural rigor in perinatal care, as advanced by Dr. Karen Scott and others [4]. Finally, policy change is needed to facilitate doula care for marginalized communities. Insurance companies, including private and Medicaid payers, must support doula care to optimize their patients’ outcomes. Insurance companies must be flexible to identify the best mechanisms (ex: expanded Medicaid benefits vs. new policy) and include diverse doulas with a variety of training backgrounds—for example, grandfathering in experienced doulas with alternative certifications [33]. Doula care is an incredibly useful and patient-centered tool for improving birth outcomes and maternal-infant health equity by increasing patient knowledge and empowerment, advocating for patient needs and concerns, providing sociocultural safety, improving birth experiences, reducing medical intervention, and—ultimately–reducing the risk of pregnancy-related disability and death [10,13]. As maternal mortality continues to increase in the US, and more severely in the aftermath of overturning abortion access, aiding Black and other doulas of color to reach pregnant people in their communities is more critical than ever [34].
